# L-asparaginase Production by *Leucosporidium scottii* in a Bench-Scale Bioreactor With Co-production of Lipids

**DOI:** 10.3389/fbioe.2020.576511

**Published:** 2020-12-17

**Authors:** Ignacio S. Moguel, Celina K. Yamakawa, Adalberto Pessoa, Solange I. Mussatto

**Affiliations:** ^1^Novo Nordisk Foundation Center for Biosustainability, Technical University of Denmark, Kongens Lyngby, Denmark; ^2^Department of Biochemical and Pharmaceutical Technology, School of Pharmaceutical Sciences, University of São Paulo, São Paulo, Brazil

**Keywords:** L-asparaginase, oleaginous yeast, lipid accumulation, bioreactor, process optimization

## Abstract

L-asparaginase (ASNase) is a therapeutical enzyme used for treatment of acute lymphoblastic leukemia. ASNase products available in the market are produced by bacteria and usually present allergic response and important toxicity effects to the patients. Production of ASNase by yeasts could be an alternative to overcome these problems since yeasts have better compatibility with the human system. Recently, it was found that *Leucosporidium scottii*, a psychrotolerant yeast, produces ASNase. In order to advance the production of ASNase by this yeast, the present study aimed to select suitable process conditions able to maximize the production of this enzyme in a bench-scale bioreactor. Additionally, the accumulation of lipids during the enzyme production process was also determined and quantified. Experiments were carried out with the aim of selecting the most appropriate conditions of initial cell concentration (1.0, 3.5, and 5.6 g L^–1^), carbon source (sucrose and glycerol, individually or in mixture) and oxygen transfer rate (*k_*L*_a* in the range of 1.42–123 h^–1^) to be used on the production of ASNase by this yeast. Results revealed that the enzyme production increased when using an initial cell concentration of 5.6 g L^–1^, mixture of sucrose and glycerol as carbon source, and *k_*L*_a* of 91.72 h^–1^. Under these conditions, the enzyme productivity was maximized, reaching 35.11 U L^–1^ h^–1^, which is already suitable for the development of scale-up studies. Additionally, accumulation of lipids was observed in all the cultivations, corresponding to 2–7 g L^–1^ (32–40% of the cell dry mass), with oleic acid (C_18__:__1_) being the predominant compound (50.15%). Since the L-asparaginase biopharmaceuticals on the market are highly priced, the co-production of lipids as a secondary high-value product during the ASNase production, as observed in the present study, is an interesting finding that opens up perspectives to increase the economic feasibility of the enzyme production process.

## Introduction

L-asparaginase (ASNase) is an important therapeutic enzyme used for treatment of acute lymphoblastic leukemia. Its mechanism of action is the inhibition of the growth of tumor cells by depriving them from nutrition. Healthy cells are able to synthesize the amino acid L-asparagine, which is necessary for its cellular functions, because they have the enzyme asparagine synthetase (EC 6.3.5.4). Tumor cells lack the enzyme asparagine synthetase, being not able to synthesize L-asparagine for their maintenance and growth. As an alternative, tumor cells take L-asparagine from the bloodstream. The administration of ASNase, promotes the hydrolysis of L-asparagine into aspartic acid and ammonia, causing, as a consequence, a decrease of L-asparagine levels in the bloodstream, leading the tumor cells to starvation and death (Brumano et al., 2019).

Currently, the ASNase available in the market is produced by bacteria (*Escherichia coli* and *Erwinia chrysanthemi*) and usually presents allergic response and important toxicity effects to the patients (Narta et al., 2007). To overcome this problem, research has been done with a PEGylated form of the enzyme, for example (Hacker, 2009). Another alternative would be finding new microorganisms able to produce ASNase with less side effects, from which, yeasts have attracted great interest since they have better compatibility with the human system (Cachumba et al., 2016). In addition, ASNase produced by yeast presents better stability in the serum and optimum pH near to the physiological conditions (Costa et al., 2016).

Yeasts isolated from extreme environment conditions have attracted particular attention for biotechnological application since they have developed special mechanisms to survive under these conditions. Psychrophile and psychrotolerant microorganisms, for example, have changed the type and production of enzymes to minimize the negative effects of low temperature. As a consequence, their proteins are resistant to cold denaturation and their enzymes show higher activity (Margesin and Schinner, 1994). In addition, psychrotolerant yeasts have shown capacity to produce valuable metabolites such as hydrolytic enzymes, pigments, polyunsaturated fatty acids, polyols, antifreeze proteins, and single cell oils (Spencer et al., 2002; Brizzio et al., 2007; Alcaíno et al., 2015), being of interest for numerous applications. Although these interesting characteristics, strains from such environments have been poorly investigated so far.

*Leucosporidium scottii*, the yeast used in this study, is an example of psychrotolerant strain able to thrive in relatively extreme environmental conditions such as low temperature, high salinity and high concentration of aromatic compounds. This yeast has been isolated from Polar Regions or temperate regions during cold weather seasons, and from Antarctic habitats of seawater, soils, mosses and saline lakes (Summerbell, 1983; Sampaio, 2011). *L. scottii* has been reported to have ability to metabolize alternative carbon sources such as xylose and glycerol, being able to produce lipases and coenzymes Q9 and Q10 (Duarte et al., 2013) as well as to accumulate lipids (Pereyra et al., 2014).

Previous experiments in our laboratory revealed that *L. scottii* is able to produce ASNase (unpublished). Since there are few studies on the production of ASNase by yeasts, this finding opens up new perspectives for the development of a new method for the production of ASNase, potentially with better characteristics than the enzyme currently available in the market, which is produced by bacteria. Based on it, efforts were done in the present study to select appropriate conditions for cultivation of this strain in a bioreactor in order to maximize the production of ASNase by this yeast. The initial concentration of cells, the carbon source (sucrose and glycerol, individually or in mixture), and the volumetric oxygen transfer coefficient (*k_*L*_a*) required for cultivation were assessed and the most suitable conditions were selected. To complete this study, the accumulation of lipids during the process for enzyme production was also determined and quantified. This is the first time L-asparaginase produced by *L. scottii* is produced and optimized in a bioreactor.

## Materials and Methods

### Microorganism and Inoculum Preparation

*Leucosporidium scottii* L115, a psychrotolerant yeast isolated from marine sediment of Antarctic Peninsula during the austral summer (2009 and 2010), was the microorganism used in this study. The strain was provided by the Culture Collection (CRM-UNESP) of the Biosciences Institute of UNESP (Rio Claro, Brazil). Stock cultures of the yeast were maintained on Yeast Malt broth (YM) with glycerol (20% v/v), at −80°C.

For inoculum preparation, a stock culture was activated in 250 mL baffled shake flasks containing 50 mL of YPD medium (dextrose 20 g L^–1^, peptone 20 g L^–1^, and yeast extract 10 g L^–1^), which were kept in an orbital shaker (New Brunswick^TM^ Innova^®^ 42) at 15°C, 200 rpm for 72 h. After this time, the cells were harvested by centrifugation (3,400 *g*, 15 min, 5°C), washed with deionized water and then cultivated in 100 mL of the same cultivation medium in 500 mL baffled shake flasks, at 15°C, 200 rpm for 48 h, in order to produce enough biomass to inoculate the bioreactors. The composition of the cultivation medium for the experiments in bioreactor was previously optimized (unpublished data) and consisted of (g L^–1^): sucrose, 28.34; glycerol, 15.61; L-proline, 6.15; KCl, 0.52; MgSO_4_.7H_2_O, 0.52; CuNO_3_.3H_2_O, 0.001; ZnSO_4_.7H_2_O, 0.001; FeSO_4_.7H_2_O, 0.001, in phosphate buffer 50 mM, pH 7.0.

### Bioreactor Experiments

Batch cultivations were performed in a benchtop multi-bioreactor system Biostat^®^ Qplus (Sartorius Stedim, Germany) equipped with automatic monitoring and control units for temperature, pH, aeration, and agitation. The 1-L bioreactor vessels were mounted with two six/bladed Rushton turbines. All experiments were performed with 0.5 L of initial working volume, pH controlled at 7.0 and temperature controlled at 15°C. Antifoam silicone based was added through syringe when the foam was nearly the top of the vessel. During the cultivations, samples were withdrawn regularly in intervals of approx. 6 h and immediately frozen at −20°C for further analysis.

#### Effect of Carbon Source

Sucrose and glycerol were used as carbon source individually (43.94 g L^–1^) or in mixture (sucrose, 28.34 g L^–1^; glycerol, 15.61 g L^–1^). Concentration values were selected in our previous study in shake flasks (unpublished results). In the present study, experiments were planned in order to verify the profile of carbon source consumption in 1-L bioreactor scale, as well as to understand the effect of sucrose and glycerol combination on ASNase production. Experiments were done in duplicate using the cultivation medium as described in section “Microorganism and Inoculum Preparation,” except by changing the carbon source. Dissolved oxygen (DO) was controlled using the cascade feature, which consists of a series of steps in which the stirrer speed and the gas mixture increase automatically to ensure that the concentration of soluble oxygen in the medium remains constant at a minimum of 30% of air saturation by controlling the agitation between 200 and 720 rpm, at constant air flow of 0.22 L min^–1^.

#### Effect of Initial Cell Concentration

In this step, cultivations were carried out using three different initial cell concentrations (1.0, 3.5, and 5.6 g L^–1^) in order to evaluate the influence of this variable on ASNase production by *L. scottii* in bioreactor. Experiments were done in duplicate using the cultivation medium as described in section “Microorganism and Inoculum Preparation.” DO was controlled at 30% of air saturation using the cascade feature through agitation (200–720 rpm) at constant air flow of 0.22 L min^–1^.

#### Effect of Volumetric Oxygen Transfer Coefficient (*k_*L*_a*)

The volumetric oxygen transfer coefficient, *k_*L*_a* (h^–1^), which is one of the most important process parameters in bioreactor operations, was also evaluated in order to maximize the ASNase production by *L. scottii* in bioreactor. The *k_*L*_a* is a parameter that represents the intensity of oxygen transfer in different bioreactors and can be calculated by the equation $\frac{dC}{dt}=k_{L}a.\left(C_{s}-C\right)$, where C_*s*_ is the value of dissolved oxygen concentration in the saturation (mg L^–1^ or g m^–3^) and C is the value of dissolved oxygen concentration in the liquid medium and varies throughout the cultivation (mg L^–1^ or g m^–3^). In this step, the experiments were carried out according to a 2^2^ Central Composite Design (CCD) with three replicates in the central point, with the aim of evaluating different combinations between the variables agitation and aeration, which led to different *k_*L*_a* values. The limits applied for the variables in these experiments (real and (coded) values) were 77.5 rpm (−1.41), 150 rpm (−1), 325 rpm (0), 500 rpm (+1), 572.5 rpm (+1.41) for agitation; and 0.02 L min^–1^ (−1.41), 0.10 L min^–1^ (−1), 0.30 L min^–1^ (0), 0.50 L min^–1^ (+1), 0.58 L min^–1^ (+1.41) for airflow. The correspondent values of *k_*L*_a* were determined experimentally using the method of “gas out-gas in” (Tribe et al., 1995) at the same condition of the experiments, taking into account the volume and media composition. Statistical analysis and response surface methodology were used to analyze the individual and interactive effects of agitation and airflow on the responses of ASNase (U L^–1^) and lipids accumulation (g L^–1^).

### Analyses

#### ASNase Activity

Most microorganisms produce ASNase intracellularly in the periplasmic, cytoplasmic, and membrane bound (Kumar et al., 2011). In the case of yeasts, a number of important enzymes are located and active in the periplasm space. In order to extract ASNase from the yeast periplasmic space, the hydroxylaminolysis method was used, which consists in a quantification of the aspartic β-hydroxamate produced by the hydroxylaminolysis reaction in the presence of hydroxylamine (Frohwein et al., 1971). According to this method, a periplasmic activity of ASNase can be quantified directly in the whole cell without previous extraction. For analysis, the cells were harvested by centrifugation (5,000 *g*, 15 min) and washed twice with deionized water. After that, cell pellets were suspended to OD_600_ of 1.0 using 25 mM Tris-HCl buffer pH 8. Then, 0.8 mL of cell suspension was mixed with 0.1 mL of 0.1 M L-asparagine and 0.1 mL of 1.0 M hydroxylamine hydrochloride solution at pH 7, in a microtube. The mixture was stirred in a thermomixer at 850 rpm, 37°C, for 30 min. Afterward, 0.25 mL of ferric solution (100 g L^–1^ FeCl_3_, 50 g L^–1^ trichloroacetic acid in 0.66 M HCl) was added. The reaction mixture was centrifuged (3,400 *g*, 4°C, 5 min) and the absorbance of the supernatant was measured at 500 nm using a microplate reader (Synergy MX, Biotek). Blanks were prepared at the same manner but using water instead of the cell suspension. One unit (U) of ASNase was defined as the amount of enzyme able to produce 1 μmol of β-aspartohydroxamic acid per minute at 37°C per gram of dried cell weight (U g^–1^). This value was further expressed as U L^–1^ of medium.

#### Cell and Substrate Quantification

Samples taken during the cultivations were centrifuged at 5,000 *g* for 5 min. Then, the supernatants were used for substrate (sucrose and glycerol) quantification while the harvested cells were washed twice with deionized water and dried at 60°C until constant weight to determine the cell dried weight (Li and Mira de Orduña, 2010). Glycerol concentration was quantified by high-performance liquid chromatography using a Biorad^®^ HPX 87H column (300 × 7.8 mm) at 60°C, 5 mM H_2_SO_4_ as mobile phase in a flow rate of 0.6 mL min^–1^, and RI detector at 50°C. Sucrose, glucose and fructose concentrations were determined using the enzymatic assay kit K-SUFRG (Megazyme) according to the manufacturer’s protocol (K-SUFRG 06/14).

#### Process Parameters

The substrate to cell conversion yield (*Y_*X/S*_ −* g_*cell*_ g^–1^_*s*__*ubstrate*_) was calculated as the ratio between cell production and substrate (glucose and/or glycerol) consumption. Volumetric productivity of cells (*Q_*X*_ −* g_*cell*_ L^–1^ h^–1^) was calculated as the ratio between cell production and cultivation time. The maximum specific growth rate (μ*_*max*_*− h^–1^) was determined as the slope of linear region on an *ln* (*X/X_0_*) versus time plot, where *X* (g L^–1^) was the cell concentration in dry cell weight per volume and *X*_0_ (g L^–1^) was the cell concentration at the initial time (0 h). Substrate to product conversion yield (*Y_*P/S*_ −* g_*product*_ or U_*enzyme*_ g^–1^_*s*__*ubstrate*_) was calculated as the ratio between the ASNase or total lipids production and substrate (glucose and glycerol) consumption. Volumetric productivity of product (*Q_*P*_ −* g_*product*_ or U_*enzyme*_ L^–1^ h^–1^) was calculated as the ratio between ASNase or total lipids production and cultivation time (h). The yield in product formation per cell (*Y_*P/X*_ −* g_*product*_ or U_*enzyme*_ g^–1^_*c*__*ell*_*)* was calculated as the ratio between product and cells. The specific rates (μ− h^–1^) were calculated by the ratio between volumetric productivity (*Q*− g L^–1^ h^–1^) and cell concentration (g L^–1^) at the correspondent time (h).

#### Lipids Extraction, Quantification, and Compositional Analyses

A quantitative method was used for total lipids determination and compositional analyses. At the end of the cultivation, the cells were recovered by centrifugation (5,000 *g*, 5 min), washed twice with deionized water and suspended to 10–30 g L^–1^ in deionized water. Then, 2 mL of this suspension were centrifuged (5,000 *g*, 5 min). Supernatant was discharged and 500 μL of glass beads (0.22 mm) and 500 μL of deionized water were added followed by homogenization using a homogenizer Precellys 24 (Bertin Technologies) during 10 cycles of 6,000 rpm for 20 s each, with a period of cooling down in ice between each cycle of 10 s. The homogenized cells were recovered by centrifugation (10,000 *g*, 5 min, 4°C) and washed once with methanol (10,000 *g*, 5 min, 4°C).

Extraction of lipids from homogenized cells was performed according to the method described by Bligh and Dyer (1959) with modifications, using a reduced amount of solvent (30 mL of methanol and chloroform, 1:1). Lipids quantification, expressed as percentage, was determined gravimetrically after evaporation of chloroform. Extracted lipids were used for preparation of fatty acid methyl esters using the American Oil Chemists’ Society [AOCS] (1998a). C_23__:__0_ methyl ester was used as an internal standard. Fatty acid methyl esters (FAME) were analyzed using the Agilent 7890A GC system equipped with a DB-WAX column (10 m × 0.1 mm × 0.1 μm Agilent Technologies), using the American Oil Chemists’ Society [AOCS] (1998b). Fatty acids were expressed as percent of total fatty acids form C_8_ to C_24_.

### Statistical Analysis

Statistical analysis of the experimental data was carried out using the software Statistica version 10.0 (StatSoft, Inc., Tulsa, OK, United States). The analysis of variance (ANOVA) and *p*-values were obtained at 90% confidence level.

## Results and Discussion

### Effect of Carbon Source on ASNase Production

**FIGURE 1:**
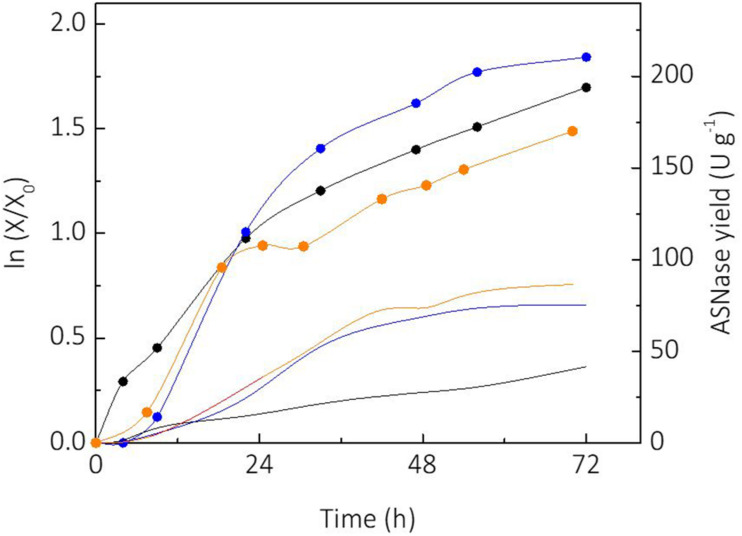
Comparison of linearized growth using different carbon sources (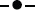 glycerol; 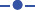 sucrose; 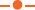 sucrose + glycerol), and specific L-asparaginase (ASNase) production per cell using different carbon sources (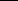 glycerol; 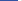 sucrose; 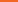 sucrose + glycerol).

Sucrose and glycerol were tested as carbon sources for cultivation of *L. scottii*. Sucrose is a substrate widely used for industrial biotechnology processes due to the well-established sugar industry (Marques et al., 2015). On the other hand, consumption of glycerol by microorganisms represents an alternative for use of this side product from the biodiesel industry, which has made efforts to convert it into value-added products during the last years (Klein et al., 2017). Figure 1 shows the growth of *L. scottii* [expressed as linearized growth (*ln (X/X_0_*))] and the ASNase yield (ASNase produced per cell) from the different substrates studied. As can be seen, the yeast growth rate was faster when sucrose was used as sole carbon source, compared to glycerol as sole carbon source or sucrose + glycerol mixture. However, the ASNase yield from sucrose + glycerol mixture was higher than from sucrose or glycerol as sole carbon source. The increased production of ASNase per cell from mixture of sucrose and glycerol indicates a mechanism of synergy of the sugars over the yeast’s growth. In this case, the highest specific growth rate was achieved on sucrose (0.068 h^–1^), while the lowest was achieved on glycerol (0.038 h^–1^). It is interesting to note that the growth profile in mixture of sucrose and glycerol presented two clear stages with different growth rates: an initial stage until 25 h with μ*_*max*_* of 0.063 h^–1^, and another stage from 30 h with μ*_*max*_* of 0.014 h^–1^ that corresponded to the exhaustion of sucrose and starting of glycerol consumption. The shifting of carbon source reduced the specific growth rate of the yeast to a value lower than that observed when glycerol was used as sole carbon source, which indicates a growth stress factor. Such stress factor caused an overexpression of ASNase production genes, which resulted in a better yield of ASNase.

Another relevant aspect observed in the experiment from sucrose, was the production of a small amount of glycerol (3.6 g L^–1^) during the cultivation. Such fact could not be observed in the other cultivation media since glycerol was used as a carbon source in those cases. It has been reported that the production of glycerol by yeasts occurs under stress situation in order to assist the osmoregulation (Flores et al., 2000). This would explain the capacity of *L. scottii* to survive in marine environment where intracellular osmotic regulation plays a key role due to the hypertonic characteristics of the sea. Glycerol synthesis has been reported during the cultivation of other yeasts including *Debaryomyces hansenii, Saccharomyces cerevisiae*, *Zygosaccharomyces rouxiii* and marine yeasts to counterbalance the external osmotic stress due the salt concentration, expressing specific genes involved to control the levels of compatible solutes (Reed et al., 1987). Glycerol is produced by reduction of the glycolytic intermediate dihydroxyacetone phosphate to glycerol 3-phosphate (G3P) followed by a dephosphorylation of G3P to glycerol. In this pathway, specific genes are induced by hyperosmotic stress, deficiency of oxygen, and also oxidative stress (Påhlman et al., 2001). Moreover, glycerol production is essential for the growth of yeast cells during reduced water availability (Albertyn et al., 1994; Rigoulet et al., 2004). This could explain the ability *of L. scottii* to metabolize glycerol.

With regard to sucrose metabolism and sugars preference, *L. scottii* showed a mechanism similar to the common yeast *S. cerevisiae*, in which sucrose breakdown occurs by the expression of a hydrolase (invertase or another fructofuranosidase) in the periplasmic space being the monosaccharides subsequently absorbed. Glucose is the preferred carbon source of many types of yeast since it represses the transcription of genes necessary for the metabolism of other carbon sources. As a consequence, alternative carbon sources are consumed only after its depletion (Gancedo, 1998; Flores et al., 2000). This pattern was confirmed in the experiments discussed in the next session.

### Influence of Initial Cell Concentration on ASNase Production

**FIGURE 2:**
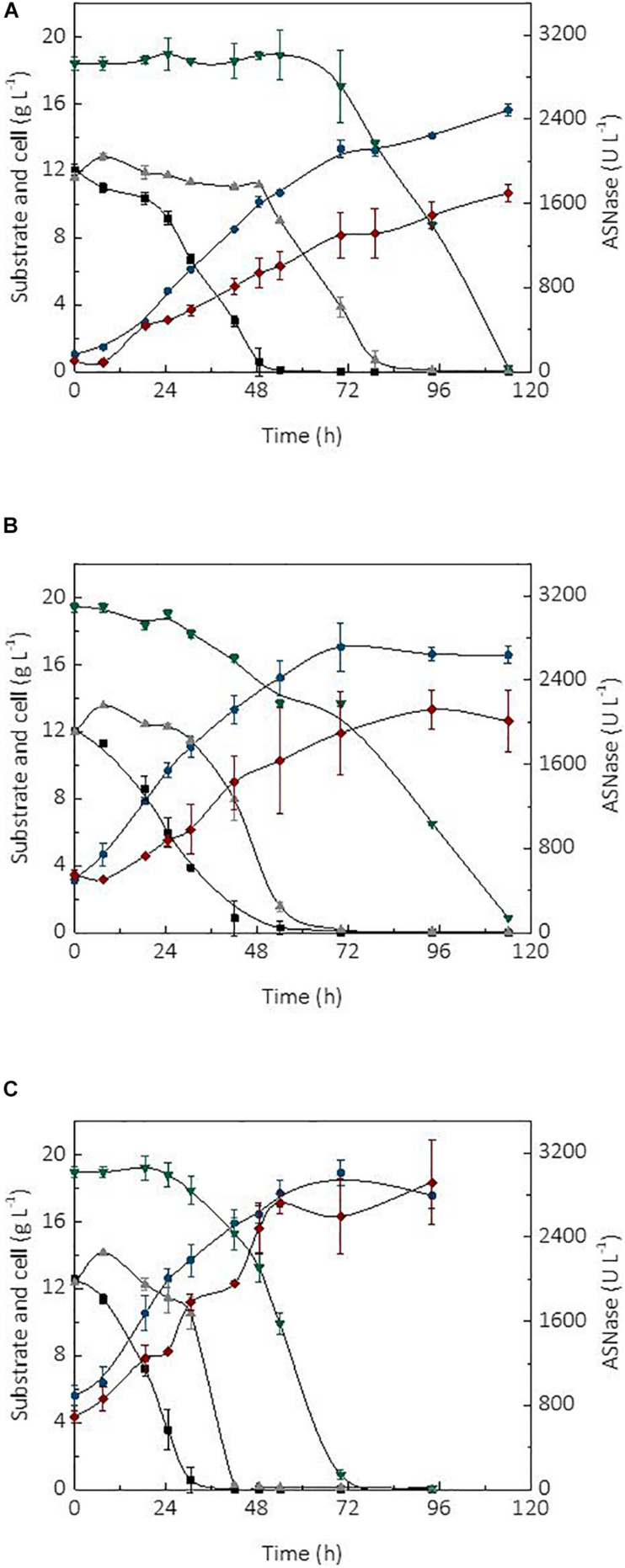
Kinetic profile of carbon source consumption, L-asparaginase (ASNase) and cell production during the cultivations of *Leucosporidium scottii* in bioreactor under different initial cell concentrations *X*_0_: **(A)** 1 g L^–1^, **(B)** 3.5 g L^–1^, **(C)** 5.6 g L^–1^. 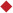 ASNase; 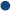 cell; 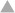 fructose; 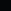 glucose; and 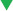 glycerol.

The initial cell concentration or inoculum size is an important parameter affecting the performance of a bioprocess (Serrano et al., 2012). In fact, the use of high cell densities combined with the type of bioreactor and mode of operation, are factors able to significantly improve the productivity of a process (Chang et al., 2014). In turn, high productivity is essential for the robustness and scale-up of a production process as it may cause a reduction of operational costs and capital investment. The kinetic profile of substrate (sucrose and glycerol) consumption, cell growth, and ASNase production during the cultivation of *L. scottii* in a bioreactor using different initial cell concentrations (1, 3.5, and 5.6 g L^–1^) is shown in Figure 2. In all cultivations, the ASNase production was associated with growth of the strain. This is a relevant information for the establishment of a cultivation strategy to asset the enzyme production through minimal growth and minimal nutrient requirement for cell maintenance.

Regarding the substrate, after 7.5 h of cultivation, sucrose was no longer detected in the media, suggesting that *L. scottii* quickly inverted this sugar into glucose and fructose. Among the different carbon sources, glucose was preferentially consumed by the yeast, followed by fructose and glycerol. All sugars were consumed within 114 h of cultivation when an initial cell concentration of 1.0 or 3.5 g L^–1^ was used, and this time was reduced to 70 h for an initial cell concentration of 5.6 g L^–1^ (Figure 2). In fact, increasing the initial cell concentration from 1.0 to 5.6 g L^–1^ caused a reduction of the maximum specific growth rate (μ*_*max*_*) nearly by half (0.068–0.035 h^–1^), while the substrate consumption rate (*Q_*S*_)* was increased from 0.40 to 0.65 g L^–1^ h^–1^ (Table 1). The production of ASNase was also higher when more cells were used to start the cultivations, resulting in an increase of the enzyme productivity (*Q*_*P*_) from 13.90 to 26.64 U L^–1^ h^–1^, with an increase in the enzyme production yield per cell (*Y*_*P/X*_) from 111.51 to 143.70 U g^–1^. Moreover, the specific substrate consumption rate (μ*_*S*_*) and specific ASNase production rate (μ*_*P*_*) increased noticeably when the number of cells was increased to 5.6 g L^–1^. It is important to highlight that the cell growth in all experiments was limited by the quantity of proline (nitrogen source) used in the assays, which was the same amount (6.15 g L^–1^) for all the initial cell concentrations studied. For this reason, all the conditions resulted in a similar value of *Y*_*X/S*_ (Table 1). This nutrient limitation could explain the increase of ASNase efficiency represented by μ*_*P*_* from 0.905 to 1.407 U g_*cell*_^–1^ h^–1^, which can be related to the yeast stress mechanism for cellular maintenance. The higher the initial cell concentration, the less the nitrogen available per cell, which caused a stress competition with consequent increase of ASNase production.

**TABLE 1:** Process parameters obtained during the production of L-asparaginase by *Leucosporidium scottii* in bioreactor under conditions of controlled dissolved oxygen at 30% of air saturation, using different initial cell concentrations (*X*_0_).

					Substrate		Cell			ASNase			
*X*_0_	Time	μ _*max*_	Cell_*final*_	ASNase_*final*_	*Q*_*S*_	μ _*S*_	*Y*_*X/S*_	*Q*_*X*_	μ _*X*_	*Y*_*P/S*_	*Y*_*P/X*_	*Q*_*P*_	μ _*P*_
g L^–^^1^	h	h^–^^1^	g L^–^^1^	U L^–^^1^	g L^–^^1^ h^–^^1^	g g_*cell*_^–^^1^ h^–^^1^	g g^–^^1^	g L^–^^1^ h^–^^1^	h^–^^1^	U g^–^^1^	U g^–^^1^	U L^–^^1^ h^–^^1^	U g_*cell*_^–^^1^ h^–^^1^
1.0	114	0.068	15.6	1,699 ± 85	0.40	0.026	0.31 ± 0.02	0.12	0.008	34.59 ± 2.93	111.51 ± 10.23	13.90 ± 0.74	0.905
3.5	114	0.047	16.7	2,012 ± 292	0.41	0.024	0.28 ± 0.02	0.11	0.007	30.28 ± 8.12	107.82 ± 30.86	12.38 ± 1.91	0.731
5.6	70	0.035	18.9	2,595 ± 359	0.65	0.034	0.29 ± 0.03	0.19	0.010	40.96 ± 10.24	143.70 ± 45.11	26.64 ± 5.12	1.407

These results indicate that the strategy of high cell density significantly intensified the ASNase production by *L. scottii*. ASNase production by a recombinant *Pichia pastoris* was also improved to 800 U g^–1^ when the cultivation was performed with a high cell density (77 g L^–1^) (Ferrara et al., 2006). The use of high cell density contributes with important advantages for project design, since it influences the capital investment by means of a reduction in the size of equipment (bioreactors, centrifuges, and filtration systems) and their facilities. In addition, a high cell density process is able to minimize side effects for the microorganisms (such as osmotic pressure, inhibition by elevated concentration of substrate or co-products) and reduce the production costs (due to the use of reduced power for mixing, heating and cooling systems). Moreover, an additional advantage related to processes for enzyme production with high cell densities is that, after the enzyme recovery, the cell biomass can be exploited as nutrient source for diverse applications.

#### Effect of *k_*L*_a* on ASNase Production

Another key parameter affecting the performance of a bioprocess is the volumetric oxygen transfer coefficient (*k_*L*_a*). Selecting an appropriate air/oxygen supply is fundamental for a successful performance of the microbial strain during the cultivation (Kerssemakers et al., 2020). In addition, the *k_*L*_a* value used in bench-scale bioreactors offers accurate information for the design, development and scale-up of a bioprocess (Garcia-Ochoa and Gomez, 2009), besides being an important point for studies on techno-economic assessment of a process. Since *L. scottii* is a strictly aerobic microorganism (Watson and Arthur, 1976), knowledge of the optimum range of *k_*L*_a* that benefits ASNase production in bench-scale bioreactor is of high importance.

Table 2 summarizes the values of the process parameters obtained during the cultivation of *L. scottii* in bioreactor under different *k_*L*_a* conditions. Cell concentration, ASNase and lipids production were calculated based on the final values at 72 h of cultivation discounted the initial values. Under low *k_*L*_a* values, below 23 h^–1^ (assays 1, 5, and 7), limited oxygen was available for cell respiration as the percentage of DO (dissolved oxygen) started at 75–90% and finished almost at zero. Otherwise, under the highest *k_*L*_a* value (123 h^–1^, assay 4) the process started at 94% DO and finished at 72%. However, there is a specific range of *k_*L*_a* values [88.92 h^–1^ (assay 6) and 91.72 h^–1^ (assay 8)] that benefited the ASNase production, resulting in an ASNase yield of about 53 g g^–1^ and productivity of 36 U L^–1^ h^–1^ (Table 2). Under these conditions, DO initiated at 94–98% and finished at 91–92%, revealing that the ASNase production by *L. scottii* requires fully aerobic condition during the cultivation. Moreover, when compared with the previous results obtained with an initial cell concentration of 1.0 and 3.5 g L^–1^, cultivated at 30% of air saturation, the ASNase productivity increased significantly from 14 to 27 U L^–1^ h^–1^ (Table 1) up to 36 U L^–1^ h^–1^ under fully aerobic condition (Table 2), which corresponded to an yield of 200 U per gram of cell. Regarding the substrate to cell conversion yield (*Y*_*X/S*_), assays 6 and 8 presented values of 0.27 and 0.28 g g^–1^, respectively, similar to the results observed in the previous experiments. It can be concluded that higher ASNase yield was correlated to higher cell yield since the enzyme production was associated to the growth. Moreover, the lowest values of *Y*_*X/S*_, which were observed for the assay 1 (the lowest *k_*L*_a* value (1.42 h^–1^) here studied), confirm that *L. scottii* is a strict aerobic microorganism.

**TABLE 2:** Process parameters obtained during the cultivation of *Leucosporidium scottii* in bioreactor under different *k_*L*_a* conditions.

								Cell		ASNase			Lipids		
Assay	Agitation	Airflow	*k_*L*_a*	μ _*max*_	Cell_*final*_	ASNase_*final*_	Lipids	*Y*_*X/S*_	*Q*_*X*_	*Y*_*P/S*_	*Y*_*P/X*_	*Q*_*P*_	*Y*_*P/S*_	*Y*_*P/X*_	*Q*_*P*_
	rpm	L min^–^^1^	h^–^^1^	h^–^^1^	g L^–^^1^	U L^–^^1^	g L^–^^1^	g g^–^^1^	g L^–^^1^ h^–^^1^	U g^–^^1^	U g^–^^1^	U L^–^^1^ h^–^^1^	g g^–^^1^	g g^–^^1^	g L^–^^1^ h^–^^1^
1	150 (−1)	0.1 (−1)	1.42	0.028	5.9 ± 0.2	523 ± 54	1.86 ± 0.26	0.13 ± 0.01	0.06 ± 0.01	13.78 ± 0.65	105 ± 7	6.68 ± 0.36	0.05 ± 0.01	0.41 ± 0.05	0.03 ± 0.01
2	150 (−1)	0.5 (+1)	74.11	0.046	12.4 ± 0.3	1,893 ± 75	4.94 ± 0.20	0.25 ± 0.02	0.15 ± 0.01	38.04 ± 2.65	170 ± 10	25.39 ± 1.31	0.10 ± 0.01	0.42 ± 0.02	0.07 ± 0.01
3	500 (+1)	0.1 (−1)	41.75	0.035	13.4 ± 0.3	1,880 ± 123	5.99 ± 0.01	0.24 ± 0.02	0.16 ± 0.01	37.82 ± 2.52	161 ± 10	25.19 ± 1.30	0.12 ± 0.01	0.51 ± 0.08	0.08 ± 0.01
4	500 (+1)	0.5 (+1)	123.0	0.053	13.7 ± 0.3	1,880 ± 82	5.80 ± 0.33	0.26 ± 0.02	0.17 ± 0.01	38.66 ± 2.52	147 ± 10	25.53 ± 1.30	0.12 ± 0.01	0.46 ± 0.03	0.08 ± 0.01
5	78 (−1.4)	0.3 (0)	4.19	0.025	6.8 ± 0.3	772 ± 41	1.93 ± 0.02	0.20 ± 0.01	0.08 ± 0.01	27.09 ± 1.10	133 ± 9	10.08 ± 0.53	0.07 ± 0.01	0.35 ± 0.01	0.03 ± 0.01
6	572 (+ 1.4)	0.3 (0)	88.92	0.057	14.5 ± 0.4	2,613 ± 267	4.25 ± 1.11	0.27 ± 0.02	0.21 ± 0.01	53.10 ± 3.73	197 ± 11	35.64 ± 1.81	0.09 ± 0.02	0.33 ± 0.08	0.06 ± 0.01
7	325 (0)	0.02 (−1.4)	23.25	0.041	14.3 ± 0.5	2,062 ± 131	4.44 ± 0.95	0.26 ± 0.02	0.18 ± 0.01	40.47 ± 2.76	154 ± 10	28.60 ± 1.43	0.09 ± 0.02	0.34 ± 0.07	0.06 ± 0.01
8	325 (0)	0.6 (+1.4)	91.72	0.046	14.3 ± 0.5	2,583 ± 261	4.96 ± 0.46	0.28 ± 0.02	0.17 ± 0.01	53.13 ± 3.57	202 ± 13	35.11 ± 1.79	0.10 ± 0.01	0.40 ± 0.04	0.07 ± 0.01
9	325 (0)	0.3 (0)	53.21	0.044	12.7 ± 0.5	2,179 ± 207	5.43 ± 0.33	0.24 ± 0.02	0.15 ± 0.01	34.11 ± 2.23	143 ± 5	22.70 ± 1.51	0.12 ± 0.01	0.49 ± 0.03	0.08 ± 0.01
10	325 (0)	0.3 (0)	53.21	0.044	17.62 ± 0.5	2,057 ± 179	6.99 ± 0.60	0.26 ± 0.02	0.17 ± 0.01	41.22 ± 2.77	163 ± 11	27.42 ± 1.42	0.12 ± 0.01	0.45 ± 0.03	0.08 ± 0.01
11	325 (0)	0.3 (0)	53.21	0.044	17.52 ± 0.4	1,828 ± 112	5.41 ± 1.18	0.20 ± 0.01	0.14 ± 0.01	32.22 ± 2.16	169 ± 12	24.66 ± 1.26	0.12 ± 0.01	0.47 ± 0.04	0.07 ± 0.02

**FIGURE 3:**
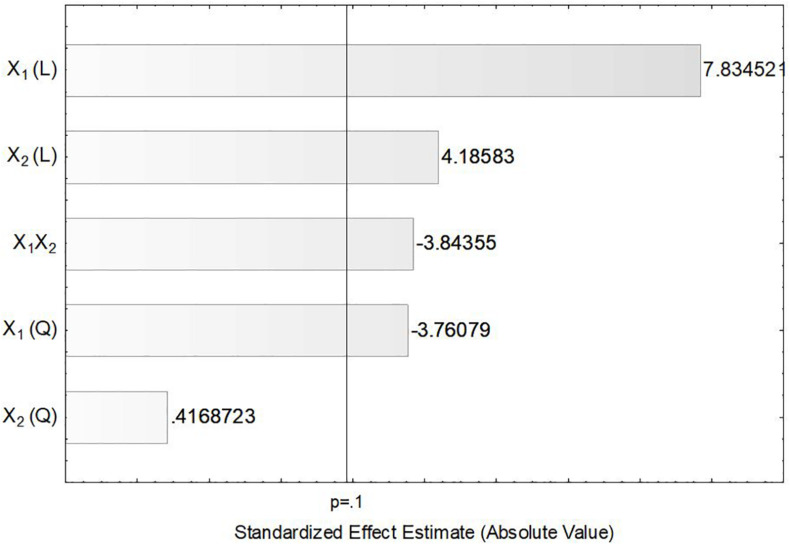
Pareto chart for estimation of the effects of agitation (*X*_1_) and aeration (*X*_2_) on the production of L-asparaginase by *Leucosporidium scottii* in a bench-scale bioreactor.

In order to verify the individual effects of agitation and aeration on the production of ASNase, a Pareto chart was plotted (Figure 3) in which, bars extending beyond the vertical line correspond to effects statistically significant at 90% confidence level. This figure clearly shows that the linear effects of agitation (*X*_1_) and aeration (*X*_2_), as well as their interaction *X_1_X_2_* and the quadratic effect of *X*_1_ had significant influence on the production of ASNase by *L. scottii*. Overall, the response was improved by increasing the agitation and aeration used during the cultivation (positive values in the Pareto chart), with the effect of the agitation being the most relevant. The significance of the quadratic term suggests that the maximum value of agitation evaluated in this study is already in an optimum region.

**FIGURE 4:**
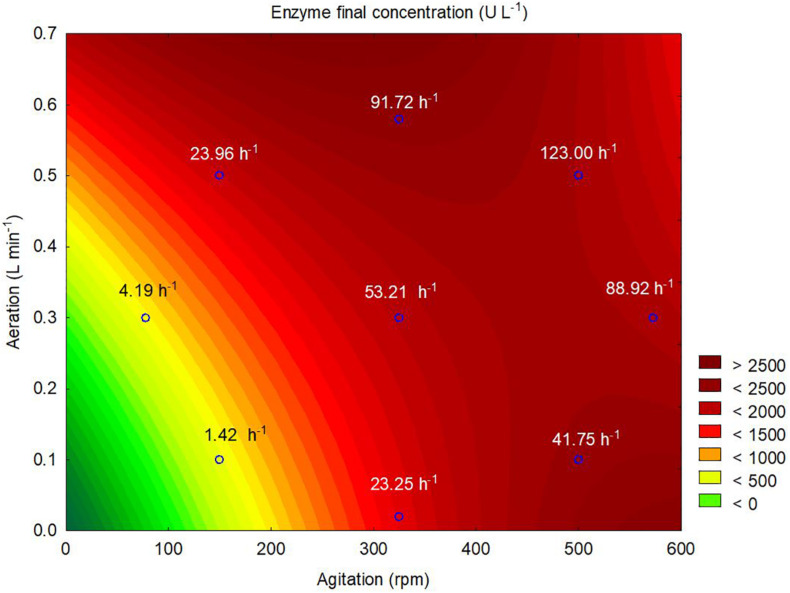
Contour plot representing the effects of the agitation and aeration on the production of L-asparaginase by *Leucosporidium scottii* in a bench-scale bioreactor. Values inside the graph represent the *k_*L*_a* resulting from the combination between agitation and aeration to each tested condition.

According to the contour plot presented in Figure 4, there are two regions where the production of ASNase is maximum (higher than 2,000 U L^–1^): one at *k_*L*_a* of 91.72 h^–1^ that corresponded to aeration and aeration of 325 rpm and 0.58 L min^–1^, respectively, and other at *k_*L*_a* of 41.75 h^–1^ that corresponded to the use of 500 rpm and 0.1 L min^–1^ during the cultivation. For industrial implementation, the power input by the impellers is a point of concern due to mechanical and capital costs and possibility of cell damage by shear forces (Noorman et al., 2018). Therefore, the *k_*L*_a* of 91.72 h^–1^, which resulted from a combination of lower agitation and higher aeration, was selected as optimum for ASNase production. Under this condition, ASNase was produced with an activity of 2,583 U L^–1^, yield of 53.13 U g^–1^ and productivity of 35.11 U L^–1^ h^–1^. These values compare very well to others reported in the literature for different ASNase producer yeasts including *Candida dublineensis* (1,040 U L^–1^) and *Candida kefyr* (1,160 U L^–1^) (Vimal and Kumar, 2017).

#### Lipids Accumulation by *Leucosporidium scottii*

Oleaginous microorganisms can accumulate intracellular triglycerides in more than 20% of biomass weight, with a fatty acid composition similar to vegetable oils. For example, the oleaginous yeast *Yarrowia lipolytica* YB-392 has been reported to accumulate up to 40% of lipids [mainly composed by oleic acid (C18:1), palmitic acid (C16:0) and linoleic acid (C18:2)], when cultivated in medium with a carbon to nitrogen (C:N) ratio of 100 (Liu et al., 2020). Similarly, *L. scottii* was previously reported to accumulate 32% of lipids when cultivated in glycerol medium with a C:N ratio of 57 (Pereyra et al., 2014). In fact, the carbon and nitrogen sources as well as their ratio, and the oxygen supply, are important factors affecting the accumulation of lipids in yeasts.

**FIGURE 5:**
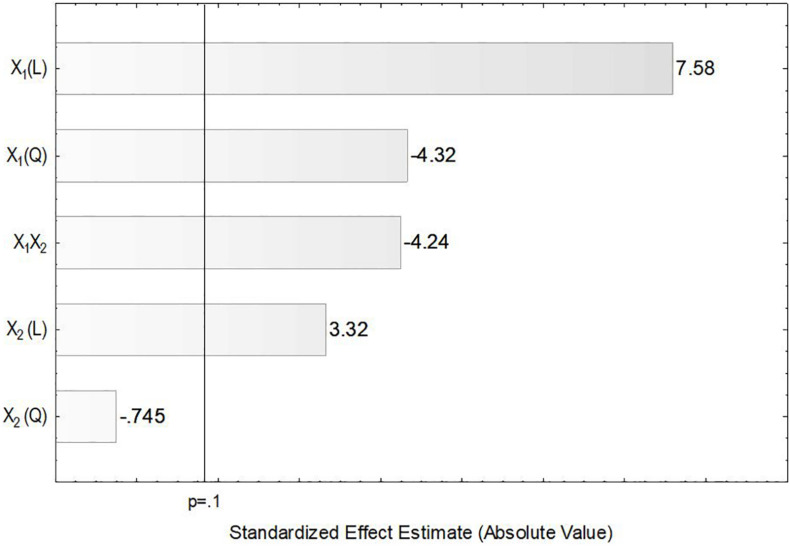
Pareto chart for estimation of the effects of agitation (*X*_1_) and aeration (*X*_2_) on lipids accumulation by *Leucosporidium scottii* in a bench-scale bioreactor.

In the present study, the accumulation of lipids by *L. scottii* was determined at the end of all the cultivations carried out for evaluation of the *k_*L*_a*. In these experiments, the carbon source and the C:N ratio (17.15) were the same for all the cultivations, only the *k_*L*_a* value was different. As can be seen in Table 2, lipids were accumulated in all the cultivations, but varied in a range between 2 and 7 g L^–1^ (corresponding to 32–40% of the cell dry mass). The conversion yield of substrate into lipids, *Y*_*P/S*_, varied from 0.05 to 0.12 g g^–1^, indicating a low lipids yield (16–38%) based on the theoretical value of 0.32 g g^–1^ (Jin et al., 2015). However, this yield of lipids could be improved by increasing the C:N ratio up to 120, for example, through an increase of sugar concentration to approx. 97 g L^–1^ (Braunwald et al., 2013). It is worth remembering that the cultivation conditions used in the present study were defined to promote high production of ASNase, while lipids were obtained as secondary products. However, similar to what was observed for ASNase, the increase of lipids accumulation per gram cell, *Y*_*P/X*_, was also dependent on the combination of agitation and aeration used during the cultivation. As can be seen in the Pareto chart in Figure 5, the effect of the variables on lipids accumulation were very similar to the effects observed on ASNase production (Figure 3), with significant and positive effects of the linear terms of both variables, agitation and aeration, as well as their interaction *X_1_X_2_* and the quadratic term of *X*_1_. This means that the highest accumulation of lipids by *L. scottii* occurred in the same region where the ASNase activity was maximum. This is an interesting result since in a global perspective, lipids could be obtained as a secondary high-value product during the production of ASNase by *L. scottii*, which could increase the economic feasibility of the production process.

The composition of fatty acids produced by *L. scottii* during the cultivation in bioreactor is summarized in Table 3. The main unsaturated fatty acids produced were oleic acid (C_18__:__1_) (50.15%) followed by linoleic acid (C_18__:__2_) (14.88%) and traces of long-chain polyunsaturated fatty acids (C_20_ and C_22_, 3.44%); while the main saturated fatty acids included palmitic acid (C_16__:__0_) (16.72%) followed by stearic acid (C_18__:__0_) (8.56%). Overall, the composition of fatty acids produced by *L. scottii*, containing oleic acid as the predominant compound, is similar to that reported by *Rhodosporidium toruloides* from glucose based medium, which included oleic acid (C_18__:__1_) (54.39%) followed by palmitic acid (C_16__:__0_) (21.67%), stearic acid (C_18__:__0_) (17.89%), and linoleic acid (C_18__:__2_) (3.25%) (Liu et al., 2021); and to that reported by *Rhodotorula glutinis* also from glucose based medium, which included 30–44% of oleic acid (C_18__:__1_), 27–42% of linoleic acid (C_18__:__2_), 6–24% of palmitic acid (C_16__:__0_), 4–9% of stearic acid (C_18__:__0_), and 4–7% of linolenic acid (C_18__:__3_) (Braunwald et al., 2013).

**TABLE 3:** Composition of fatty acids produced by *Leucosporidium scottii* during the cultivation in bench-scale bioreactor.

Fatty acids	Mean value* (%)
Myristic acid (C_14:0_)	0.61 ± 0.08
Palmitic acid (C_16:0_)	16.72 ± 1.68
Palmitoleic acid (C_16:1_)	0.53 ± 0.21
Stearic acid (C_18:0_)	8.56 ± 2.51
Oleic acid (C_18:1_)	50.15 ± 4.40
Linoleic acid (C_18:2_)	14.88 ± 1.59
Octadecatrienoic acid (C_18:3 *n*__–__4_)	4.46 ± 1.09
Arachidic acid (C_20:0_)	0.54 ± 0.11
Gadoleic acid (C_20:1 *n*__–__11_)	0.23 ± 0.06
Arachidonic acid (C_20:4 n__–__6_)	0.10 ± 0.06
cis-11-docosenoic acid (cetoleic) (C_22:1 n__–__11_)	0.96 ± 0.42
Docohexanoic acid (DHA) (C_22:6 n__–__3_)	1.61 ± 0.95

*Mean value corresponds to the average values considering all the cultivations.

The composition of fatty acids produced by *L. scottii* would be suitable for application on the production of biodiesel for example, since in this case, the presence of large amount of the mono unsaturated oleic acid and trace amount of poly unsaturated fatty acids are the most desirable. Among the list of biodiesel specification according to ASTM (American Society for Testing and Materials), cloud point (CP) is significantly important for biodiesel originated from vegetable oils. The cloud point is the temperature at which the first solids appears. Biodiesel fuel originated from soybean methyl esters presents CP of approximately (0°C), while the biodiesel fuel originated from rapeseed/canola methyl esters possesses CP of approximately −3°C. The difference in fatty acid profile of the C_18__:__1_ and C_18__:__2_ caused this CP improvement. Soybean oil contains 21–24% C_18__:__1_ and 49–53% C_18__:__2_, whereas rapeseed/canola oil contains 58–62% C_18__:__1_ and 21–24% C_18__:__2_ (Knothe, 2009), which are more similar to the values obtained for *L. scottii* in the present study.

## Conclusion

This study reported for the first time the production of the enzyme L-asparaginase by the yeast *Leucosporidium scottii* in a bioreactor. The enzyme production was significantly improved by selecting appropriate conditions of initial cell concentration (5.6 g L^–1^), *k_*L*_a* of 91.72 h^–1^, and a mixture of sucrose and glycerol as carbon source, reaching a productivity of 35.11 U L^–1^ h^–1^. Accumulation of lipids was also observed during the cultivations for enzyme production, which is an interesting result as lipids are high-value compounds with important industrial applications and may, therefore, contribute to the economic feasibility of the enzyme production process. The findings of this study open up new opportunities for the production of the therapeutic enzyme L-asparaginase with perspectives of resulting in an enzyme with less side effects (produced by yeast instead of bacteria) and more economically feasible (with co-production of other valuable compounds during the cultivation). Moreover, cell protein after lipids recovery, which may also be produced in significant amount in high cell density cultivations, could also be used as animal feed, maximizing the profit.

It is important to highlight that L-asparaginase is an antileukemic biopharmaceutical that has been used worldwide. However, the products on the market are highly immunogenic because they are produced by bacteria, procaryotic microorganisms that irritate the patient’s immune system. As a consequence, all current medications cause severe side effects and great suffering to patients. The production of L-asparaginase by a yeast (eukaryotic microorganism), as prosed in the present study, has potential to be less immunogenic since human cells are also eukaryotic and should be less visible to anti-asparaginase antibodies. By selecting the appropriate process conditions, an enzyme productivity of 35.11 U L^–1^ h^–1^ was obtained in the present study, which is high enough for the development of scale-up studies. Moreover, it is worth mentioning that the L-asparaginase biopharmaceuticals on the market are highly priced (ranging from US$400 to US$5,000 per dose) and the development of a process with the generation of other associated products, as suggested in the present study, has great potential to reduce the cost of the final product.

## Data Availability Statement

The raw data supporting the conclusions of this article will be made available by the authors, without undue reservation, to any qualified researcher.

## Author Contributions

IM: investigation and analysis. CY: investigation, analysis, and writing—original draft. AP: supervision and resources. SM: supervision, resources, writing—review, editing, and project administration. All authors contributed to the article and approved the submitted version.

## Conflict of Interest

The authors declare that the research was conducted in the absence of any commercial or financial relationships that could be construed as a potential conflict of interest.
